# Synthesis, Characterization, Antibacterial, Antifungal and Anticorrosion Activities of 1,2,4-Triazolo[1,5-a]quinazolinone

**DOI:** 10.3390/molecules28145340

**Published:** 2023-07-11

**Authors:** Walid Ettahiri, Rajae Salim, Mohamed Adardour, Elhachmia Ech-chihbi, Ismaeel Yunusa, Mohammed M. Alanazi, Sanae Lahmidi, Azeddin El Barnossi, Oussama Merzouki, Abdelilah Iraqi Housseini, Zakia Rais, Abdesselam Baouid, Mustapha Taleb

**Affiliations:** 1 Laboratory of Engineering, Electrochemistry, Modeling and Environment, Faculty of Sciences, Sidi Mohamed Ben Abdellah University, Fez 30000, Morocco; 2 Laboratory of Molecular Chemistry, Faculty of Sciences Semlalia, Cadi Ayyad University, Marrakech 40001, Morocco; 3 College of Pharmacy, University of South Carolina, Columbia, SC 29208, USA; 4 Department of Pharmaceutical Chemistry, College of Pharmacy, King Saud University, Riyadh 11541, Saudi Arabia; 5 Laboratory of Heterocyclic Organic Chemistry, Department of Chemistry, Faculty of Sciences, Mohammed V University in Rabat, Rabat 10000, Morocco; 6 Laboratory of Biotechnology, Environment, Agri-Food and Health, Faculty of Sciences Dhar El Mahraz, Sidi Mohammed Ben Abdellah University, Fez 30000, Morocco

**Keywords:** Triazolo[1,5-a]quinazolinone, biological activity, molecular docking, corrosion inhibition, adsorption

## Abstract

The synthesis of 5,6,7,8-tetrahydro-[1,2,4]triazolo[5,1-b]quinazolin-9(4H)-one (THTQ), a potentially biologically active compound, was pursued, and its structure was determined through a sequence of spectral analysis, including ^1^H-NMR, ^13^C-NMR, IR, and HRMS. Four bacterial and four fungal strains were evaluated for their susceptibility to the antibacterial and antifungal properties of the THTQ compound using the well diffusion method. The impact of THTQ on the corrosion of mild steel in a 1 M HCl solution was evaluated using various methods such as weight loss, potentiodynamic polarization (PDP), electrochemical impedance spectroscopy (EIS), and scanning electron microscopy (SEM) analysis. The study revealed that the effectiveness of THTQ as an inhibitor increased with the concentration but decreased with temperature. The PDP analysis suggested that THTQ acted as a mixed-type inhibitor, whereas the EIS data showed that it created a protective layer on the steel surface. This protective layer occurs due to the adsorption behavior of THTQ following Langmuir’s adsorption isotherm. The inhibition potential of THTQ is also predicted theoretically using DFT at B3LYP and Monte Carlo simulation.

## 1. Introduction

**Figure 1:**
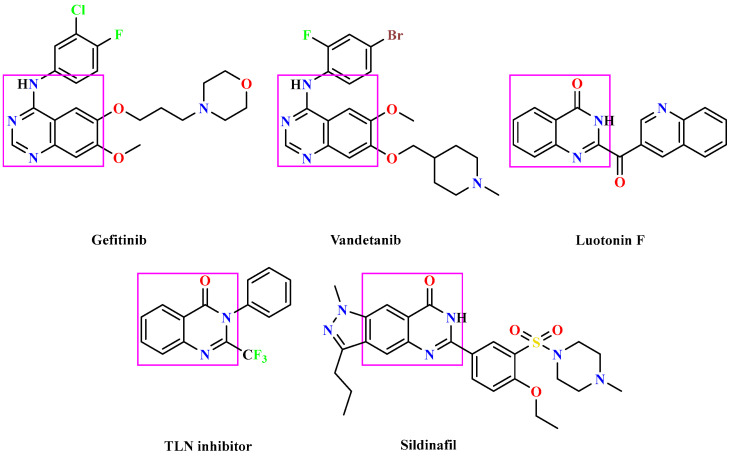
Examples of biologically active triazolo-quinazolines.

Compounds containing nitrogen heterocycles make up a significant portion of small drug molecules that have been approved by the Food and Drug Administration (FDA), amounting to approximately 60% [1]. Numerous new nitrogen heterocycles have been synthesized for their applications in biological and material sciences [2,3,4,5,6,7,8,9,10]. Quinazoline is a frequently occurring structural feature in natural products and pharmaceutically active molecules. Triazole-fused quinazolinones, which are particularly significant in pharmaceuticals, contain fused quinazolines (Figure 1) [11,12,13,14]. These compounds possess a range of useful biological properties, including anti-SARS-CoV-2 [14,15,16], anticancer [17,18,19], antiviral [20,21,22], antimicrobial, anti-inflammatory [23,24,25,26], antimalarial, and antifungal activities [27,28,29,30]. The drug development process can result in the elimination of many potential candidates due to pharmaco-kinetic issues, leading to a significant investment of time and resources. To mitigate this, the focus was on synthesizing triazolo-quinazoline through an eco-friendly and simple condensation process without the use of catalysts or bases. The objective of this research was to investigate the biophysical characteristics of a newly created triazolo-quinazoline compound and assess its potential as an antibacterial and antifungal agent. Additionally, molecular docking simulations were used to analyze the binding positions and interactions between the synthesized molecules and their target receptors [31].

The economy and safety of metals can be significantly affected by corrosion. To reduce the detrimental effects of corrosion, the application of inhibitors is a practical solution, especially in acidic environments [32]. Inhibitors used for acidic environments often consist of organic compounds that contain heteroatoms like nitrogen, sulfur, and oxygen, and also possess π bonds [33,34,35,36,37,38]. Multiple factors can influence the adsorption of organic inhibitors on a metal surface, such as the inhibitor type, the surface charge of the metal, the corrosive environment’s nature, and the interaction between the metal surface and the inhibitor. In an acidic medium like hydrochloric acid, the metal surface carries a positive charge due to its dissolution, while the organic inhibitor, when protonated, also becomes positively charged [39]. Additionally, the results of this study are compared with the effects of other inhibitors of the same category in order to identify the most efficient inhibitors in terms of anti-corrosion behavior. The advancement of new and more effective inhibitors for carbon steel in acidic environments remains a positive impact on the materials and chemical industries, particularly in situations where corrosion protection is crucial. Moreover, the use of environmentally friendly and low-cost inhibitors, such as THTQ in the present work, helps to reduce the environmental impact and minimize the cost of corrosion protection. This study seeks to provide a comprehensive assessment of the anti-corrosive characteristics of THTQ on mild steel in a 1 M HCl solution. This includes analyzing its inhibition mechanism, effectiveness, and mode of action. The results of this research will serve as a valuable reference for future investigations focused on the development of corrosion inhibitors for mild steel in acidic environments.

## 2. Results and Discussion

### 2.1. Chemistry

**Scheme 1:**
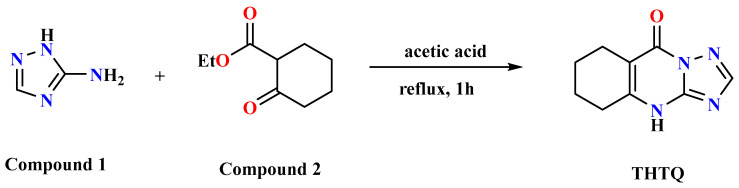
Synthesis of THTQ.

The experimental procedure was performed utilizing 0.5 g (equivalent to 5.95 millimoles) of 1H-1,2,4-triazol-5-amine (Compound 1) and 0.951 milliliters (equivalent to 5.95 millimoles) of ethyl 2-oxo-cyclohexanecarboxylate (Compound 2) as starting materials. These starting materials were combined and heated under reflux in 10 milliliters of acetic acid for 1 h. After cooling the reaction mixture at room temperature, the resulting solid was filtered, washed with acetic acid and ethanol, and then dried under vacuum (Scheme 1). This synthesized procedure is economic compared to that previously performed by Berecz et al. since the desired molecule was obtained with a small mole number and achieving a high yield of 97% in a short reaction time [40].

The obtained product was characterized by employing several spectroscopic techniques, including infrared (IR) spectroscopy, proton (^1^H) and carbon-13 (^13^C) nuclear magnetic resonance (NMR) spectroscopy, and high-resolution mass spectrometry.

The obtained compound (THTQ) is a white solid with a yield of 97% and a melting point of 262 °C. The infrared spectrum of the compound in KBr exhibited characteristic peaks at 3475 cm^−1^ for quinazoline-NH and 1610 cm^−1^ for C=O. The ^1^H NMR spectrum obtained using DMSO as a solvent displayed peaks at 1.65 ppm (multiplet, 4H, 2CH_2_-quinazoline), 2.32 ppm (triplet, 2H, CH_2_-quinazoline), 2.55 ppm (triplet, 2H, CH_2_-quinazoline), 3.66 ppm (singlet, N–H), and 8.09 ppm (singlet, C–H, triazolic). The ^13^C NMR spectrum of the compound in DMSO showed peaks at 20.75, 21.06, 21.14, and 26.34 ppm (4CH_2_-quinazoline), 105.66, 147.75, and 149.51 ppm (3C, =C-), 151.82 ppm (C–H, triazolic), and 156.33 ppm (C=O). The high-resolution mass spectrum of the [M + H]^+^ compound demonstrated a *m*/*z* value of 190.08546, which corresponds well with the calculated value of 190.09 for C_9_H_10_N_4_O.

### 2.2. Biological Results

#### 2.2.1. Antibacterial Activity Screening of Compound THTQ

Table 1 presents the results of the inhibition zone observed for the synthesized product against several bacteria. The strains tested included *Proteus mirabilis*, *Escherichia coli*, Bacillus subtilis, and Staphylococcus aureus. The inhibition zone ranged from 15 mm to 16 mm for *E. coli* and *S. aureus*, and from 9 mm to 19 mm for *B. subtilis* and *P. mirabilis*. Among the tested strains, the highest inhibition zone of 9 mm was obtained for compound THTQ against *B. subtilis*.

The compound THTQ demonstrated the ability to inhibit the growth of the majority of the bacteria tested, as presented in Table 1. It exhibited the strongest inhibitory effects against *Proteus mirabilis*, with a minimum inhibitory concentration of 1.875 mg/mL, and against *Escherichia coli*, with a MIC of 3.75 mg/mL. This could be attributed to the compound’s hydrophobic nature, as hydrophobic compounds with high partition coefficients can easily cross biological membranes. Additionally, many proteins contain hydrophobic amino acids that can interact with lipophilic molecules.

#### 2.2.2. Antifungal Activity of Compound THTQ

Table 2 summarizes the inhibition zone results of the synthesized product against *Fusarium oxysporum*, *Aspergillus niger*, *Aspergillus flavus*, and *Candida albicans*. The inhibition zone ranged from 22 mm to 42.85 mm for the fungal strains. The most potent effect of the tested product was against *Aspergillus niger*, with an inhibition zone of 28.57 mm, which was equal to that of fluconazole.

Subsequently, the antifungal activity of the synthesized compound was assessed through in vitro testing against three fungal strains: *Aspergillus niger*, *Candida albicans*, and *Aspergillus flavus*. The product THTQ is a good inhibitor against *Aspergillus niger* with an MIC value of 15 mg/mL, which is comparable to that of the standard antibiotic fluconazole. However, it only shows moderate activity against *Candida albicans* with an MIC value of 7.5 mg/mL and *Aspergillus flavus* with an MIC value of 15 mg/mL.

### 2.3. Molecular Docking

Table 3 presents the docking scores of THTQ against two enzymes, *E. coli* Topoisomerase IV and Cytochrome P450 14 α-sterol demethylase (CYP51) from Mycobacterium tuberculosis, as revealed by the obtained docking results. The ligand exhibited docking scores of −6.1 kcal/mol and −7.0 kcal/mol, respectively. In comparison, the standard compounds, streptomycin and fluconazole, exhibited docking scores of −5.3 kcal/mol and −7.0 kcal/mol, respectively. It is worth noting that these proteins have been previously studied using docking techniques [41].

**Figure 2:**
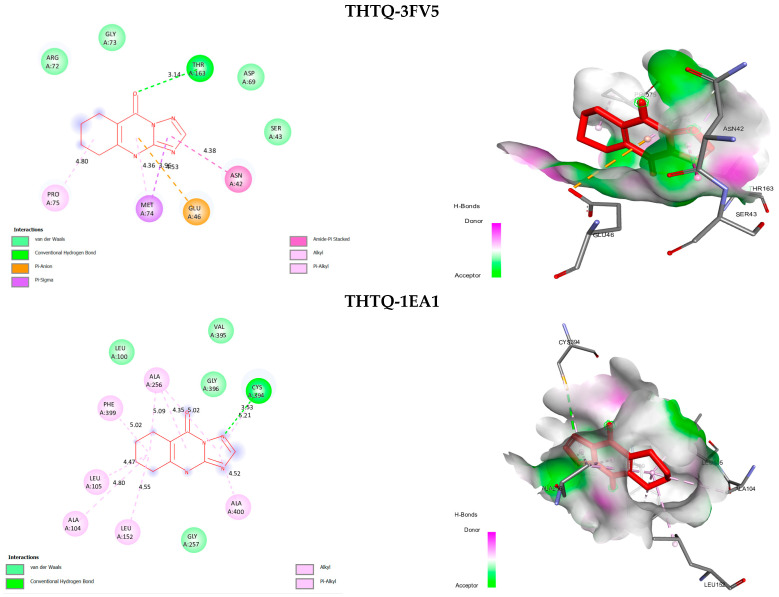
Molecular docking of THTQ with 3FV5 and 1EA1proteins, surfaces around ligand and 2D forms.

The analysis of the molecular docking with 3FV5 protein showed that THTQ has hydrophobic, electrostatic and hydrogen bond interactions in addition to van der Waals interactions. THTQ interacted with the enzyme by forming pi-anion with GLU46, a pi-sigma and pi-alkyl with MET74, an amide-pi stacked with ASN42 and alkyl with PRO75. Also, THR163 has a conventional hydrogen bond with THTQ, shown in Figure 2.

The information shown in Figure 2 indicates that THTQ interacts with the 1EA1 protein through several hydrophobic interactions such as pi-alkyl and alkyl interactions with CYS394, ALA400, LEU152, ALA104, LEU105, PHE399, and ALA256, as well as van der Waals interactions. Furthermore, THTQ forms a standard hydrogen bond with CYS394.

The data presented suggest that THTQ has a favorable binding conformation with both the *E. coli* Topoisomerase IV and the Cytochrome P450 14 α-sterol demethylase enzymes from Mycobacterium tuberculosis, indicated by its binding energy of −6.1 kcal/mol and −7.0 kcal/mol, respectively. Furthermore, THTQ interacts with several amino acid residues, including CYS394, ALA400, LEU152, ALA104, LEU105, PHE399, and ALA256, through hydrophobic interactions such as pi-alkyl and alkyl interactions, in addition to weak van der Waals interactions. Additionally, THTQ forms one conventional hydrogen bond with CYS394 in each protein.

### 2.4. Corrosion Test

#### 2.4.1. Potentiodynamic Polarization Analysis

##### Concentration Effect of the THTQ Inhibitor

**Figure 3:**
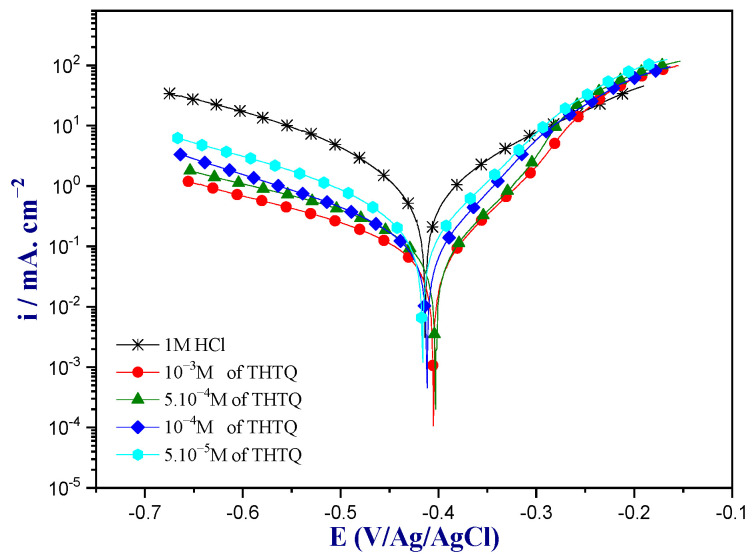
Potentiodynamic polarization curves for mild steel without and with THTQ concentration.

The corrosion of mild steel was analyzed using potentiodynamic polarization methods, examining how varying concentrations of THTQ affected the outcome. These tests were conducted at a temperature of 298 K, and their findings are depicted in Figure 3 and outlined in Table 4. Using the EC–lab program, the cathodic Tafel slope (βc), i_corr_, and E_corr_ were computed. The impact of surface charges on corrosion behavior was examined by shifting the potential ±250 mV compared to the open circuit potential [42,43,44,45].

According to the findings, when the concentration of THTQ was raised, the polarization curves obtained in the inhibited solution showed a minor decline. This was supported by a reduction in the current density (i_corr_) values, as shown in Table 4. At a concentration of 10^−3^ M, the inhibition efficiency reached as high percentage of 94.4%. Additionally, a substantial change in potential was observed in the anodic zone (from −250 mV to −150 mV), indicating that THTQ was desorbed from the working electrode’s surface. The inhibitors can be classified as either cathodic or anodic based on the displacement of E_corr_ values in the inhibited solution compared to the E_corr_ value in the uninhibited solution. The corrosion potential values showed a minor change (less than 85 mV), suggesting that THTQ can be classified as a mixed-type inhibitor. The THTQ compound was found to have a negligible effect on the cathodic slopes, suggesting that it does not significantly modify the mechanism of hydrogen reduction [39].

##### Effect of Temperature

**Figure 4:**
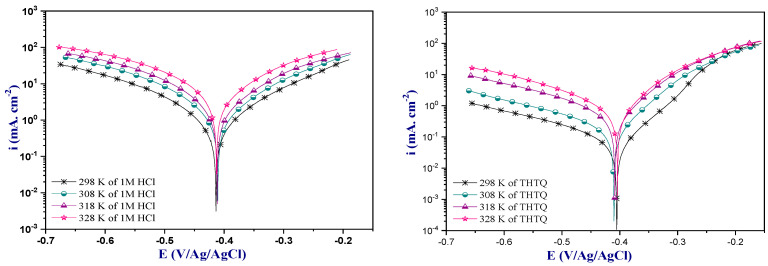
Polarization plots for mild steel with and without addition of THTQ at different temperatures.

As previously reported, the effect of temperature on the interaction between steel and an electrolyte solution is an essential factor to consider [46]. In this study, the potentiodynamic polarization curves of mild steel in a 1 M HCl were analyzed at different temperatures (ranging from 298 K to 328 K) with and without the optimal concentration of the THTQ inhibitor (10^−3^ M). The EIS diagrams were used to obtain the electrochemical parameters, which were summarized in Table 5. The current study found that as the temperature rose, the cathodic area of the inhibitor increased slightly. This observation is supported by the current densities presented in Table 5 and depicted in Figure 4.

The THTQ inhibitor was found to lower the current densities compared to the corrosion medium alone, indicating its adsorption activity. Nonetheless, a minor decline in inhibition efficiency was observed when the temperature increased from 298 K to 328 K, indicating that the inhibitor acted as good inhibitor even at high temperature [47].

The polarization curves clearly show that there is a direct correlation between an increase in temperature and an increase in current density values. This rise in current densities is more significant in the absence of inhibitors compared to their presence, which suggests that the molecules adsorbed onto the steel surface. Moreover, Table 5 displays that as the temperature increased from 298 K to 328 K, the i_corr_ values for THTQ increased from 52 µA cm^−2^ to 537 µA cm^−2^. However, despite this rise in i_corr_, the inhibition efficiency only experienced a minor reduction with the increase in temperature.

Both Arrhenius equation and its alternative formulation were employed to calculate the activation energy (Ea), activation enthalpy (ΔH*), and activation entropy (ΔS*) for the corrosion of mild steel in 1 M HCl. The values of activation energy, enthalpy, and entropy obtained through the Arrhenius equation and its alternative formulation can offer useful insights into the mechanism of corrosion inhibition. Equations (1) and (2) represent the Arrhenius equation and its alternative formulation, respectively, and were derived using the following equations [48]:

(1) $$i_{corr}=Ae^{\left(\frac{-E_{a}}{RT}\right)},$$

(2) $$i_{corr}=\frac{RT}{Nh}\;e^{\left(\frac{\Delta S^{*}}{R}\right)}\;e^{\left(\frac{-\Delta H^{*}}{RT}\right)}.$$

The variables in these equations include absolute temperature (T), Plank’s constant (h), Avogadro’s number (N), and gas constant (R).

**Figure 5:**
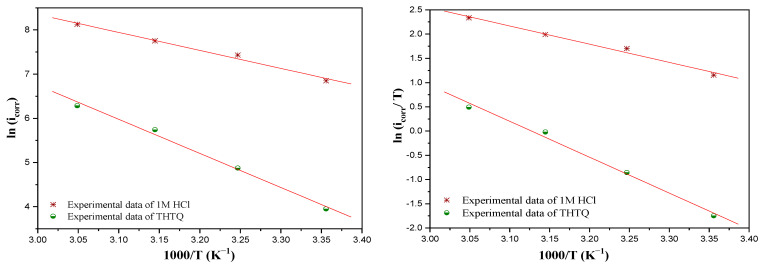
Arrhenius and transition state plots for mild steel in 1 M HCl solution with and without the optimum concentration of THTQ.

The activation energy was obtained by fitting ln(i_corr_) versus (1000/T) using linear regression, while ΔH* and ΔS* were calculated by fitting ln(i_corr_/T) versus (1000/T) (Figure 5). The R^2^ value of 0.998 ± 0.03 obtained from the plots suggests that the kinetic activation parameters were accurately calculated and highly reliable.

Based on the data presented in Table 6, it can be inferred that the activation energy (Ea) values for the inhibited solution are higher than those for the uninhibited solution. This result suggests that THTQ is likely adsorbing onto the surface of the mild steel through electrostatic bonds or physical adsorption [49]. Additionally, the positive values of activation enthalpy (ΔH*) indicate that the dissolution reaction necessitates energy in the form of heat to take place, thereby suggesting an endothermic reaction. The negative values of activation entropy (ΔS*) further suggest that the activated complex formed during the corrosion process of mild steel becomes more ordered compared to the blank solution [50].

#### 2.4.2. Electrochemical Impedance Spectroscopy of THTQ

##### Concentration Effect of THTQ

**Figure 6:**
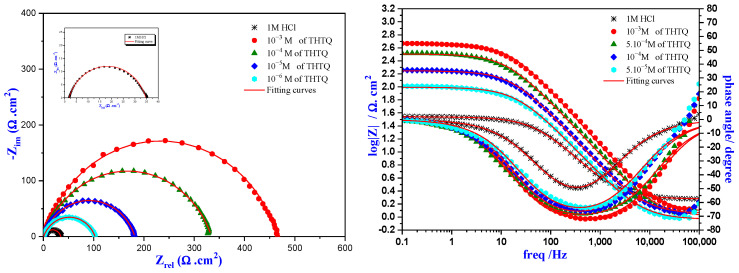
Nyquist and Bode plots for mild steel in the studied corrosive medium with and without various concentrations of THTQ at 298 K.

The objective of using the EIS method was to explore the impact of concentration and gain a more comprehensive understanding of the underlying mechanisms that cannot be observed using polarization curves alone. Thus, to examine the inhibitory action of THTQ, the EIS approach was employed. Figure 6 displays the Nyquist and Bode plots obtained for mild steel in the presence of different concentrations of THTQ, as well as in the absence of THTQ. The electrochemical parameters extracted from the EIS plots are summarized in Table 7, which was generated following a detailed simulation process.

**Figure 7:**
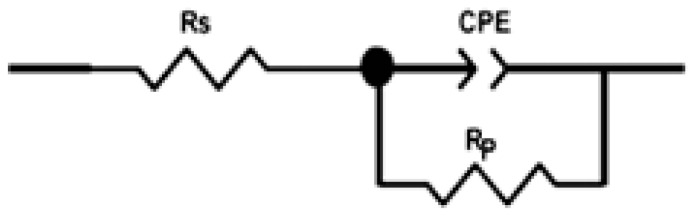
Equivalent circuit used for experimental impedance simulation.

The Nyquist plots in Figure 6 show that they do not perfectly form a semicircle. The presence of surface heterogeneity due to impurities, surface roughness, and inhibitor adsorption is a possible explanation for the deviations observed in the experimental data. In order to enhance the accuracy of the fitting, a constant phase element (CPE (n, Q)) was employed instead of a pure capacitor, as depicted in Figure 7. The resulting plots exhibited a single semicircle, indicating that the corrosion reaction mechanism is primarily controlled by charge transfer [51].

The presence of only one peak in the mid-frequency range on the Bode plot further supports the charge transfer mechanism, as does the observation of a single time constant at the interface between the metal and electrolyte for THTQ. The angle of the phase was below −90°, which matched the dip observed in the Nyquist plots, providing support for the equivalent circuit employed for simulation. Additionally, it was observed that the phase angle increased as the THTQ concentration increased, indicating a direct correlation between THTQ adsorption on the surface of mild steel and the formation of a protective layer.

The equations (Equation (3)) were used to determine the double layer capacity (C_dl_). The polarization resistance (R_p_), constant phase angle (CPE) represented by Q, and the heterogeneity values of the surface represented by n (ranging from 0 to 1) were used in the calculation [52].

(3) $$C_{dl}={\left(Q\times R_{p}^{1-n}\right)}^{1/n}.$$

The results in Table 7 demonstrate that the R_p_ values exhibited an upward trend as the THTQ concentration increased, with a value of 465 Ω cm^2^ at the optimum concentration of 10^−3^ M. On the other hand, both the constant phase element (Q) and the capacitive double layer (C_dl_) decreased slightly. Specifically, the C_dl_ for THTQ dropped from 80.1 to 25.2 µF.cm^−2^. The results indicate that the THTQ inhibitor adsorbed on the mild steel surface and formed a protective layer, which prevented the metal from contact with the HCl solution. The inhibition efficiency, which achieved 92.9% at the optimum THTQ concentration, further supports this conclusion.

##### Adsorption Isotherms of THTQ

**Figure 8:**
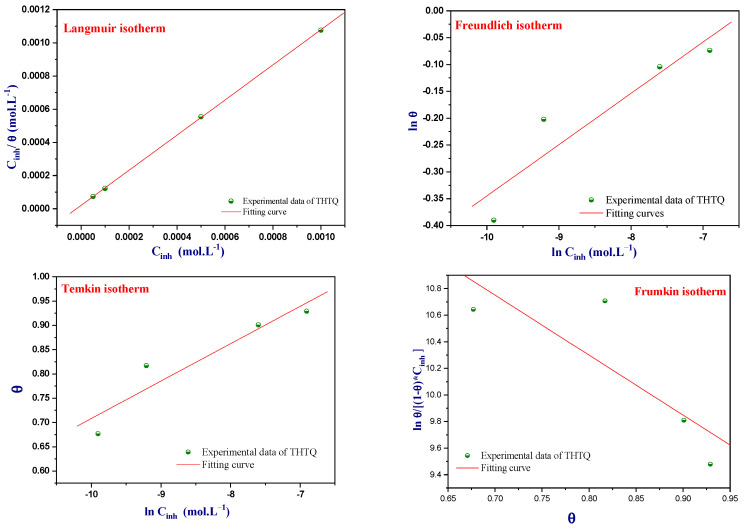
Adsorption isotherms model for THTQ at 298 K.

The investigation of adsorption isotherm models is crucial for comprehending the adsorption mechanism of inhibitors on metal surfaces. In this study, various adsorption isotherms such as Temkin, Frumkin, Langmuir, and Freundlich were employed to study the adsorption mode of THTQ on the steel sample based on the EIS results (Figure 8). The obtained parameters from the four isotherms were summarized in Table 8 to investigate the adsorption behavior of THTQ on the metal surface in a 1 M HCl.

Furthermore, researchers calculated the standard Gibbs free energy of adsorption (ΔG°_ads_) using Equation (8) [51].

The standard Gibbs free energy of adsorption (ΔG°_ads_) can be calculated using the equation that incorporates the molar concentration of water in the solution (55.5), the universal gas constant (R = 8.314 J/mol·K), and the absolute temperature (T).

(8) $$\Delta G_{\mathrm{ads}}^{o}=-\mathrm{RT}ln(55.5K).$$

Table 9 shows that the adsorption constants for both Frumkin and Freundlich isotherms were found to be insignificant, indicating that THTQ does not conform to these models, despite the R^2^ regression coefficient values being close to 1. However, parameter (a) obtained from Temkin isotherm had a negative value, which led us to suggests that there is a presence of a repulsive interaction between the adsorbed molecules.

According to previous research, when the ΔG°_ads_ value is approximately −20 kJ/mol or less, it indicates a physical adsorption mechanism between the charged inhibitor and the charged metal. Conversely, if the ΔG°_ads_ value is approximately −40 kJ/mol or higher, it suggests a chemisorption mechanism [53]. The Langmuir isotherm was used to calculate the ΔG°_ads_ values for THTQ, as it was found to be the best-fitting model for the studied molecules. As indicated in Table 9, the ΔG°_ads_ value for THTQ was determined to be −43.9 kJ/mol. This outcome suggests that the adsorption process of THTQ onto the steel surface is predominantly a chemisorption mechanism, where the inhibitor forms robust bonds with the steel [46,47,48,49,50].

#### 2.4.3. Immersed Time of THTQ

**Figure 9:**
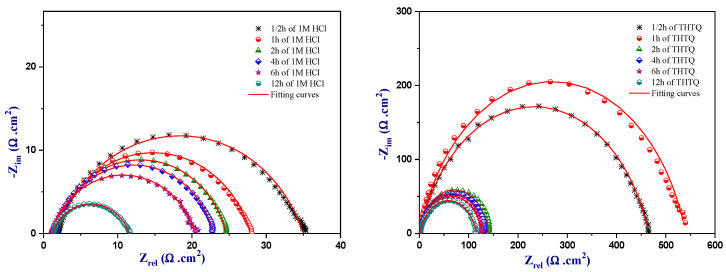
Nyquist plots for mild steel in the presence and absence of THTQ at varying immersion times.

The behavior of THTQ at different time scales was investigated using the EIS technique. This investigation was carried out on two solutions: one without the inhibitor (1 M HCl) and the other containing 10^−3^ M of the inhibitor. The samples were immersed for different periods ranging from ½ h to 12 h. The impedance diagrams are presented in Figure 9, and Table 10 provides their electrochemical parameters.

The results presented in Figure 9 indicate that the addition of THTQ produced a single, depressed semicircle loop in the Nyquist plot. As the immersion time increased, the size of the semicircle decreased, suggesting that our molecule acted as a good and effective inhibitor in the studied conditions. These observations were consistent over a range of immersion times, from ½ h to 12 h.

The results of the immersion time experiment showed a slight increase in the R_P_ value for THTQ from 465 Ω.cm^2^ to 540 Ω.cm^2^ with 1 h. At the same time, there were slight decreases in the double-layer capacitance. After this, the calculated inhibition efficiency of THTQ showed a slight decrease, which then became more pronounced. However, the inhibition efficiency percentage (ƞ_imp_%) decreased slightly until achieved 71.3% in the immersion time of 12 h. Therefore, it can be inferred that THTQ exhibited high inhibition behavior as the immersion time increased, indicating that the inhibitor molecules were adsorbed onto the steel surface to form a protective layer [54].

#### 2.4.4. Surface Analyses MEB-EDX

**Figure 10:**
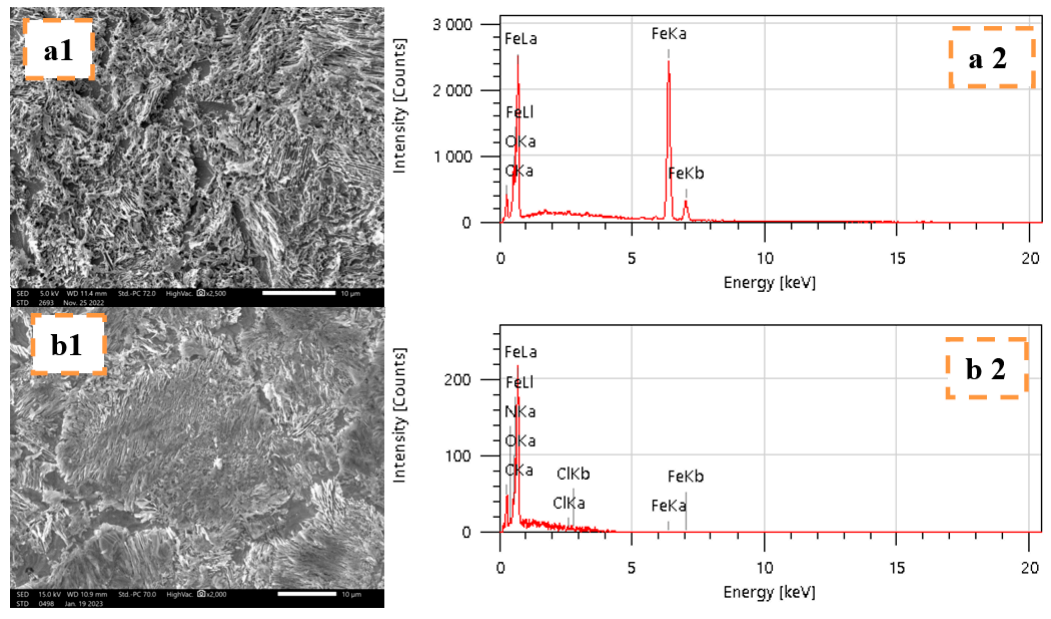
Micrograph image and EDX spectra of mild steel after immersion in 1 M HCl (**a1**,**a2**) and after immersion in 10^−3^ M of THTQ (**b1**,**b2**).

The surface morphology and composition of mild steel were analyzed after immersion in solutions with and without inhibitors for 5 h at a temperature of 298 K to investigate the nature of the adsorbed atoms. As shown in Figure 10, the surface of the sample immerged in the studied aggressive environment was affected, showing a high heterogeneity of the steel surface. Moreover, the sample that was immersed in the inhibited solution showed a non-uniform protective layer on the surface, indicating that THTQ had adsorbed on the surface of the steel and formed coordination bonds with it.

To identify the active site responsible for THTQ adsorption and to determine the chemical elements adsorbed on the mild steel surface, the EDX analysis was conducted. The analysis involved examining X-rays that were emitted during the interaction of electrons with matter. This was performed after immersing the mild steel surface in a corrosive medium with and without the THTQ inhibitor. The weight percentage results of the different elements that were found to be adsorbed on the surface of the mild steel are regrouped in Table 11.

According to the EDX microanalysis results, it appears that THTQ molecules create a protective layer on the surface of the sample, indicating their capacity to perform well when it is in contact with aggressive solutions (1 M HCl). Moreover, the detection of a low percentage of oxygen atoms implies that these atoms may participate in the interaction with mild steel. On the other sense, it can be said that the high percentage of oxygen atom in the blank solution due to the strong quantity of oxides formed at the steel samples. The detection of carbon atoms in the EDX analysis implies that the interaction between the THTQ inhibitor and mild steel may occur through this specific site, facilitating the formation of strong bonds with the iron atom d-orbitals.

#### 2.4.5. In Silico Approach Study

**Figure 11:**
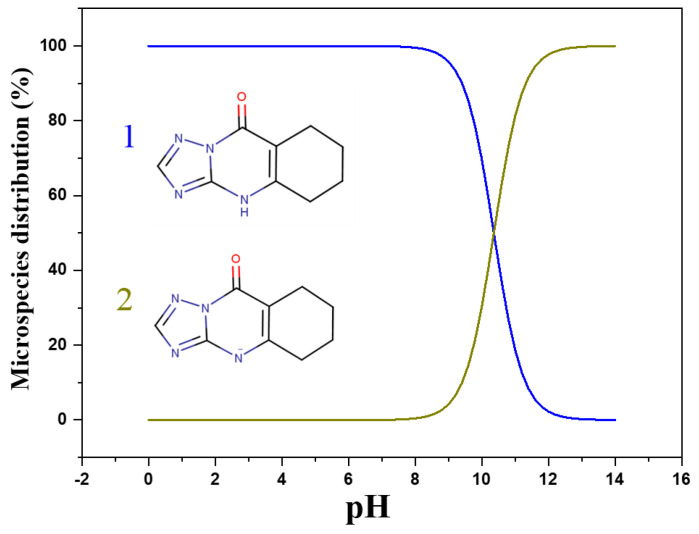
Species percentage distribution for THTQ versus pH axe.

Experimental chemists have recently acknowledged the significance of quantum chemistry in various fields of chemistry, including corrosion [55]. This statement suggests that in order to improve the accuracy of the experimental results, it is important to consider the impact of the solvent on the calculations. This can be achieved by identifying the primary protonated forms that exist in the electrolyte solution [55]. For this, the compounds under investigation were analyzed using the Marvinsketch.18 program. Figure 11 displays the prevailing form of the compounds at a pH close to zero, along with their respective distribution of protonated forms in relation to pH.

It is evident from Figure 11 that the molecule displays two distinct species, with each exhibiting a specific value. The blue curve indicates the distribution of the first molecular form (1), while the other reflect the distribution of the second form (2). The analysis of these results leads to the conclusion that the primary form of THTQ is present in the 1 M HCl solution at a pH value close to zero.

#### 2.4.6. Quantum Calculation

**Figure 12:**
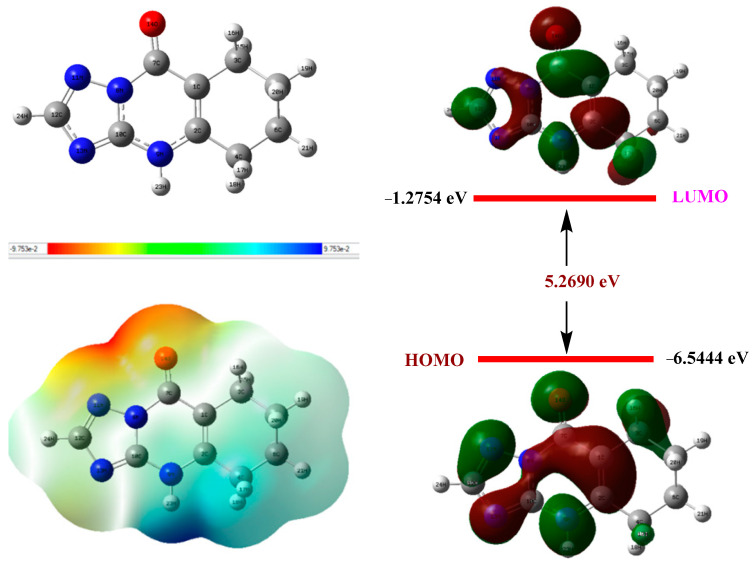
Optimized geometries, HOMO and LUMO densities, and molecular electrostatic potential (MEP) of the protonated form of THTQ.

**Figure 13:**
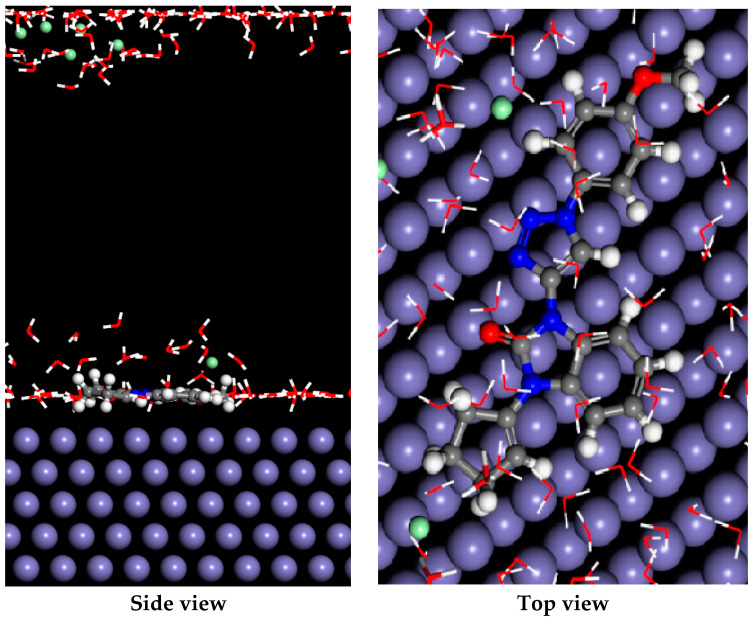
Most stable adsorption configuration of THTQ.

The theoretical methods can effectively elucidate the inhibition process in a cost-effective and efficient manner. This can be achieved by utilizing density functional theory (DFT) to calculate different chemical descriptors at the B3LYP/6–311G (d,p) basis set. As a result, various molecular descriptors such as E_HOMO_, E_LUMO_, μ, ΔE, ΔN, η, χ, and σ can be detected to understand the reactivity of the THTQ molecule. The HOMO and LUMO orbitals, as well as the electrostatic potential maps and optimized structure of the inhibitor, are presented in Figure 12. The global quantum descriptors that were calculated are listed in Table 12. The presence of red areas surrounding the oxygen and nitrogen atoms of THTQ in the molecular electrostatic potential (MEP) shown in Figure 13 suggests that THTQ was adsorbed onto the mild steel surface via these active sites. Additionally, global descriptors such as ΔE_gap_ and ΔN_110_ can provide us an insight into the molecule’s reactivity and the inhibitory performance. It is pointed out that the small gap energetic can be explained by the high reactivity of the studied molecules. In our case, the ΔE_gap_ value of THTQ equals 5.2690 eV, implying the tendency of this value to be small since THTQ shows a high inhibition performance, i.e., a high reactivity. Moreover, the obtained values of softness and hardness indicate that the THTQ molecule is more reactive since detecting a small softness value. On the other hand, it can be seen that the electronegativity index goes with the same trend of the above parameters. Furthermore, a positive value of ΔN_110_ indicates that electrons are transferred from the THTQ molecule to the metal, thereby verifying the molecule’s high reactivity performance and elucidating its adsorption onto the steel surface, which results in the formation of a protective layer [56,57,58].

#### 2.4.7. Monte Carlo Simulation Result

In order to gain insight into how the molecular structures interact with the iron surface, Monte Carlo simulation was performed on Fe 110/180 H_2_O systems. Figure 13 displays the most stable configuration of the studied inhibitor, as well as top and side views of the studied system. The extracted adsorption energies of the studied molecules regrouped in Table 13.

It can be noticed from the MC simulation that the studied molecules are adsorbed onto the iron surface in a parallel orientation, covering a significant surface area. In contrast, the adsorption energy of the water molecules is notably lower than that of the molecular structure, which can be attributed to their displacement from the iron surface. Based on the simulation results, it can be suggested that this molecular structure has the potential to form a protective layer on the surface of the sample.

## 3. Materials and Methods

### 3.1. Chemistry

The synthesis of THTQ was performed by combining 1H-1,2,4-triazol-5-amine (1) and ethyl 2-oxocyclohexanecarboxylate (2) and refluxing the mixture in acetic acid for one hour. The FT-IR spectrum of the studied products was performed by VERTEX 70-BRUKER spectrophotometer without any dilution, using KBr pellets in the range of 4000–400 cm^−1^, and with a resolution of ±4 cm^−1^ (32 scans). On the other hand, the ^1^H and ^13^C NMR spectra were carried out and recorded utilizing a Bruker Avance DPX300 spectrometer (see Experimental Parts and Supporting Information of the present paper).

### 3.2. Biological Studies

#### 3.2.1. Microbial Strains Tested

The efficiency of THTQ, the synthesized compound, against antimicrobial and antifungal activities was evaluated using four bacterial strains, namely *Escherichia coli*, *Staphylococcus aureus*, *Proteus mirabilis*, and *Bacillus subtilis*, and four fungal strains, namely *Aspergillus niger*, *Candida albicans*, *Fusarium oxysporum*, and *Aspergillus flavus*. Streptomycin and fluconazole were employed as reference drugs for antibacterial and antifungal assessments, respectively. The Minimum Inhibitory Concentration (MIC) of THTQ was evaluated.

#### 3.2.2. Diffusion Method

To assess the antimicrobial activity of THTQ, the well diffusion method was utilized [59]. To carry out the experiment, fresh crops of the four bacterial strains and *C. albicans* were inoculated onto Petri dishes containing Mueller–Hinton (MH) culture media and malt extract (ME) by employing the double-layer method. Decimal dilutions were conducted using a sterile saline solution (0.9% NaCl) until a turbidity of 0.5 McFarland (106–108 CFU/mL) was attained. Subsequently, 100 µL of the bacterial or fungal suspension was added to tubes that contained 5 mL of soft agar (0.5% agar–agar), and the tubes were then inoculated and spread onto Petri dishes containing MH and ME media using the double-layer method. Wells with a diameter of 6 mm were created at the center of the Petri dishes and then filled with 50 µL of the THTQ solution with a concentration of 30 mg/mL dissolved in 10% DMSO. The antifungal activity of THTQ against *A. flavus*, *A. niger*, and *F. oxysporum* was evaluated using the direct confrontation method in the ME medium between the THTQ compound and the fungal strains being tested. In particular, 6 mm diameter wells were formed on Petri dishes and loaded with 50 µL of the THTQ compound synthesized at a concentration of 30 mg/mL of 10% DMSO. Subsequently, a layer of agar containing the fungal strain was positioned 1 cm away from the well containing THTQ.

The use of conventional antimicrobial drugs as controls allows for a comparison of the effectiveness of THTQ to that of the existing treatments. Negative and positive controls for bacterial strains were administered with streptomycin, while fluconazole was used as a control for fungal strains. The same testing methodology applied for THTQ was employed for the control drugs. The bacterial and fungal strains were incubated in Petri dishes at 37 °C and 30 °C, respectively, for 18–24 h for bacterial strains and 1–2 days for *C. albicans*, and up to 7 days for *A. niger*, *F. oxysporum*, and *A. flavus*. The inhibition diameters and percentages were determined after the specified time points to compare the effectiveness of THTQ to that of the control drugs [60].

#### 3.2.3. Determination of the Minimum Inhibitory Concentration

The MIC of the synthesized compound THTQ was determined using the microdilution method against four bacterial strains and four fungal strains [61]. In summary, 96-well microplates were labeled in a sterile environment, and the initial column of the plate was filled with 100 µL of THTQ with a concentration of 30 mg/mL in 10% DMSO. In the remaining wells, 50 µL of sterile composite broth medium (CBM) or malt extract (ME) was added for bacterial and fungal strains, respectively. Serial dilutions were then made using a multichannel pipette, and 30 µL of microbial suspension from each strain was added to the respective wells. The microbial cultures were incubated at different temperatures and times depending on the type of microorganism. Specifically, bacterial cultures were incubated at 37 °C for 24 h, *C. albicans* for 48 h, and *A. flavus*, *A. niger*, and *F. oxysporium* for seven days at 30 °C. The minimum inhibitory concentration (MIC) was determined by observing growth in wells or by using the colorimetric method, TTC 0.2% (*w*/*v*) [62].

### 3.3. Molecular Docking

The Auto-Dock Vina 1.5.6 software was utilized for molecular docking analysis. This enabled the investigation of multiple potential modes of interaction between the ligand and the intended biological response [63]. To study the antifungal mechanism, the researchers obtained the crystal structure of the target enzyme Cytochrome P450 14α-sterol demethylase (CYP51) from Mycobacterium tuberculosis, which was bound to fluconazole (PDB ID: 1EA1), from the RCSB protein database (www.rcsb.org accessed on 14 November 2022). Similarly, to investigate the antibacterial mechanism, the crystal structure of the protein co-complexed with the inhibitor (PDB ID: 3FV5) was obtained. The protein structures were improved by adding Geister and Kollman charges, incorporating polar hydrogen atoms, and removing water molecules and heteroatoms. The resulting refined structures were then saved in a PDBQT format. The protein energy was subsequently minimized using the Auto-Dock approach, and polar hydrogen atoms were added afterwards. To prepare the ligands for molecular docking analysis, the structures of the ligands were first drawn and optimized. The optimized structures were then saved in the mol 2 format, followed by conversion of the optimized ligands to a PDBQT format for use in molecular docking analysis. In order to identify potential binding sites for the ligands, a uniform grid with a size of 40 × 40 × 40 and a spacing of 0.375 Å was generated around the active site of each protein. The grid center for *E. coli* was set at x = 12.51, y = −1.13, z = 3.26, while for Mycobacterium tuberculosis cytochrome P450 14α-sterol demethylase (CYP51), it was placed at x = −18.14, y = −6.64 and z = 66.54. The binding affinity of the ligands was measured in units of kcal/mol, with a lower value indicating a stronger binding interaction. The interactions between the protein and ligand were analyzed and displayed in both 2D and 3D using Biovia Discovery Studio Visualizer 4.0 (DSV 4.0) [41,64].

### 3.4. Corrosion Test

#### 3.4.1. Materials and Solutions

Prior to conducting each experiment, the samples utilized in the study were smoothed by employing SiC Emery paper with differing grain sizes ranging from 180 to 1500. Next, the samples underwent a cleaning process utilizing both acetone and water, and were subsequently air-dried. The samples were composed of the following elements in the indicated percentages: 99.30% Fe, 0.05% Mn, 0.09% P, 0.05% S, 0.01% Al, 0.38% Si, and 0.21% C. The concentrations of the inhibitor solutions used in the study ranged from 10^−6^ M to 10^−3^ M. To prepare a molar hydrochloric acid solution, commercial HCl (37% HCl) was diluted with distilled water.

#### 3.4.2. Electrochemical Experiments

The electrochemical experiments were carried out utilizing the Versa STAT 4 potentiostat and Versa Studio as the analysis software. A three-electrode configuration was implemented in setting up the electrochemical cell. For each experiment, the mild steel sample used as the working electrode had a surface area of 1 cm^2^. The platinum electrode served as the counter electrode and the Ag/AgCl electrode functioned as the reference electrode. Prior to each experiment, the mild steel samples were submerged in the aggressive solution and left to reach equilibrium for a duration of 30 min.

To generate Nyquist plots, an AC signal was applied at the open circuit potential with a frequency range spanning from 100 kHz to 100 mHz and an amplitude of 10 mV. Following this, an appropriate equivalent circuit was employed to simulate the obtained plots. The inhibition efficiency was subsequently calculated using Equation (9) [39].

(9) $$IE_{\mathrm{EIS}}=\frac{R_{P}^{inh}-R_{P}}{R_{P}^{inh}}\times 100,$$

where $R_{P}$ and $R_{P}^{inh}$ represent the polarization resistance in the absence and presence of the inhibitor, respectively.

The potentiodynamic polarization curves were automatically produced by sweeping the electrode potential across a range of ±250 mV relative to the open circuit potential. This was performed at a scanning rate of 1 mV/s. The inhibition efficiency (*IE_PDP_*%) was determined by utilizing the following formula (Equation (8)), where *i_corr_* is the corrosion current density of the uninhibited sample and $i_{\mathrm{corr}}^{inh}$ is the corrosion current density of the inhibited sample:

(10) $$IE_{\mathrm{PDP}}=\frac{i_{\mathrm{corr}}-i_{\mathrm{corr}}^{inh}}{i_{\mathrm{corr}}}\times 100.$$

The corrosion current density values were derived from the potentiodynamic polarization curves created throughout the electrochemical experiments [39].

#### 3.4.3. Theoretical Details

The Koopmans theorem, initially formulated in the 1930s, asserts that the negative values of the highest occupied molecular orbital (HOMO) and lowest unoccupied molecular orbital (LUMO) provide an approximation of the ionization energy and electron affinity in the ground state, respectively. This results in the following equations, based on the formulas presented by Parr and Pearson using the finite differences approach [65,66,67,68,69,70,71,72].

(11) $$\Delta E_{gap}=E_{LUMO}-E_{HOMO},$$

(12) $$\chi =\frac{1}{2}\left(E_{HOMO}+E_{LUMO}\right),$$

(13) $$\eta =\frac{1}{2}\left(E_{LUMO}-E_{HOMO}\right),$$

(14) $$\sigma =\frac{1}{\eta },$$

(15) Δεback−donation=−η4,

(16) $$\Delta N_{metal}=\frac{\Phi_{metal}-\chi_{inh}}{2\left(\eta_{metal}+\eta_{inh}\right)}=\frac{\Phi_{metal}-\chi_{inh}}{2\eta_{inh}}.$$

It is a well-established fact that the electron transfer fraction (ΔN_110_) descriptor provides us with crucial information regarding the effectiveness of corrosion inhibitors and the strength of the interaction between the inhibitor and metal surface. This variables are calculated based on the electronegativity of the inhibitor, hardness of the metal surface, and hardness of the inhibitor. The hardness value of iron is assumed to be zero, and the work function Φ value for the Fe (110) surface is 4.82 eV.

The B3LYP computational methods were carried out using the G09W package and a 6311G (d,p) basis set. The water phase simulations were performed using the Conducted Polarized Continuum Model (C-PCM) solvent model. The graphical representations were created using the Gaussview 5.0.8 software package.

#### 3.4.4. Monte Carlo Simulation Method

Monte Carlo simulation was carried out to investigate the adsorption arrangement of the examined molecular structures and their interactions with the iron surface while also accounting for the presence aggressive solution (1 M HCl) as a solvent. Prior to conducting the simulation, the components of the system under study were optimized using a COMPASS force field. The Adsorption Locator module in Materials Studio 7.0 software was then utilized to execute the simulation. The simulated system consisted of the inhibitor molecule, 180 water molecules, and a simulated sample of Fe _110_ enlarged with a supercell size of 11 × 11 and a vacuum slab of 30 A°.

#### 3.4.5. Surface Analyses

Scanning electron microscopy SEM is a potent technique employed for scrutinizing surface morphology. In this study, SEM was employed to examine the adsorption behavior of inhibitors at an immersion time of 5 h, both with and without the optimum concentration (10^−3^ M), using the QUATTRO S FEG Thermo Scientific microscope. Additionally, the material composition was assessed through energy dispersive X-ray (EDX) analysis that was attached to the SEM and functioned at an acceleration voltage of 20 kV.

## 4. Conclusions

The synthesized heterocyclic compound THTQ was identified through analysis of spectral data obtained from ^1^H, ^13^C NMR, IR, and high-resolution mass spectroscopy. Also, THTQ showed a good biological and anticorrosive activities. From the results analysis, it can be concluded that

- THTQ demonstrated a significant antibacterial and antifungal activity;
- The electrochemical impedance spectroscopy (EIS) showed a good effectiveness with a maximum inhibition efficiency of 92.9% at the optimal concentration (10^−3^ M);
- The polarization curves confirmed THTQ’s inhibition efficiency, categorizing it as a mixed-type inhibitor;
- The Langmuir isotherm model was applicable to the THTQ inhibitor, indicating the formation of a protective layer on the steel surface, as confirmed by surface analysis using MEB-EDX;
- The theoretical approach combining DFT at B3LYP and Monte Carlo simulation provided insights into the inhibition potential of THTQ.

## Figures and Tables

**Table 1:** Antibacterial activity of THTQ.

	*Staphylococcus aureus*		*Escherichia coli*		*Bacillus subtilis*		*Proteus mirabilis*	
	Antibacterial Activity (mm)	MIC (mg/mL)	Antibacterial Activity (mm)	MIC (mg/mL)	Antibacterial Activity (mm)	MIC (mg/mL)	Antibacterial Activity (mm)	MIC (mg/mL)
THTQ	15	7.5	16	3.75	9	7.5	17	3.75
	15	7.5	15	3.75	9	7.5	17	1.875
	15	7.5	16	3.75	9	7.5	19	1.875
Streptomycin	24	1.875	27	0.94	24	1.875	25	0.94
	24	1.875	27	0.94	24	1.875	25	0.94
	24	1.875	27	0.94	24	1.875	25	0.94
DMSO	Rs	-	Rs	-	Rs	-	Rs	-

**Table 2:** Antifungal activity of THTQ.

	*Candida albicans*		*Aspergillus niger*		*Aspergillus flavus*		*Fusarium oxysporum*	
	Antifungal Activity (mm)	MIC (mg/mL)	Antifungal Activity (%)	MIC (mg/mL)	Antifungal Activity (%)	MIC (mg/mL)	Antifungal Activity (%)	MIC (mg/mL)
THTQ	25	7.5	28.57	15	Rs	-	38.09	15
	22	7.5	26.19	15			38.09	15
	24	7.5	26.19	15			42.85	15
Fluconazole	20	7.5	28.57	15	Rs	-	45	7.5
	20	7.5	28.57	15			45	7.5
	20	7.5	28.57	15			45	7.5
DMSO	Rs	-	Rs	-	Rs	-	Rs	-

**Table 3:** Binding energy of the studied molecules with 3FV5 and 1EA1.

	Ligand	Biding Score (Kcal/mol)	Number Hydrogen Bond
3FV5	THTQ	−6.1	1
	Streptomycin	−5.3	7
1EA1	THTQ	−7.1	1
	Fluconazole	−7.0	3

**Table 4:** Potentiodynamic polarization parameters of mild steel in different concentrations.

Medium	Conc (M)	−E_corr_ (mV/Ag/AgCl)	i_corr_ (µA. cm^−2^)	−β_c_ (mV. dec^−1^)	β_a_ (mV. dec^−1^)	Ƞ_PP_ %
1M HCl	-	413	944	139	128	-
THTQ	10^−6^	416	226	139	79	76.0
	10^−5^	413	108	135	66	81.7
	10^−4^	403	80	132	60	91.5
	10^−3^	405	52	131	62	94.4

**Table 5:** Potentiodynamic polarization parameters of mild steel without and with THTQ at different temperatures.

Medium	Temp. (K)	−E_corr_ (mV/Ag/AgCl)	i_corr_ (µA. cm^−2^)	−βc (mV. dec^−1^)	βa (mV. dec^−1^)	Ƞ_PP_ %
1M HCl	298	413	944	139	128	-
	308	410	1690	137	129	-
	318	411	2328	126	125	-
	328	412	3387	120	133	-
THTQ	298	405	52	131	62	94.4
	308	410	131	137	64	92.2
	318	407	312	115	65	86.5
	328	405	537	117	70	84.1

**Table 6:** Thermodynamic adsorption parameters of THTQ.

Activation Parameters	Ea (kJ/mol)	∆H* kJ/mol	∆S* (J/mol·K)
1M HCl	33.86	31.26	−82.73
THTQ	64.17	−61.58	−5.11

**Table 7:** Impedance parameters for mild steel in the presence and absence of THTQ in 1 M HCl solution at 298 K.

Medium	Conc (M)	R_s_ (Ω.cm^2^)	R_p_ (Ω.cm^2^)	CPE		C_dl_ (µF.cm^−2^)	Ɵ	ƞ_imp_ %
				Q (µF.S ^n−1^)	n_dl_			
HCl	1	1.7	33.0	312.7	0.784	89.1	-	-
THTQ	10^−6^	0.40	102.3	256.8	0.759	80.9	0.677	67.7
	10^−5^	0.73	181.2	148.5	0.790	56.9	0.817	81.7
	10^−4^	0.66	333.7	106.7	0.781	41.8	0.901	90.1
	10^−3^	0.41	465.0	58.8	0.809	25.2	0.929	92.9

**Table 8:** Linear equations of isotherms used.

Isotherms	Linear Equations	Descriptions
Langmuir	$\frac{C_{\mathrm{inh}}}{\theta }=\frac{1}{K}{+C}_{\mathrm{inh}}$ (4)	K is a coefficient that represents the strength of adsorption between the inhibitor and the metal surface. C_inh_ refers to the concentration of the inhibitor used in the experiment. ϴ is the inhibitor recovery rate, which is a measure of the efficiency of the inhibitor in protecting the metal surface from corrosion.
Freundlich	$\ln\theta =\mathrm{lnK}+Z\;{\mathrm{lnC}}_{\mathrm{inh}}$ (5)	0 < Z < 1: the adsorption of inhibitor on the surface of the metal is favorable and easy. Z = 1: moderate adsorption of inhibitor on the metal surface. Z > 1: the adsorption behavior of inhibitor is difficult and less favorable.
Frumkin	$\ln\left(\frac{\theta }{\left(1-\;\theta \right)C_{\mathrm{inh}}}\right)=\mathrm{lnK}+2a\theta$ (6)	d represents the interaction between adsorbed molecules.
Temkin	$\theta =\frac{-1}{2a}\ln\left(K\right)-\;\frac{1}{2a}{\ln(C}_{\mathrm{inh}})$ (7)	a is the repulsion or attraction interaction coefficient among adsorbed compounds.

**Table 9:** Isotherm models parameters for THTQ.

Isotherms	R^2^	Parameters		K	∆G°_ads_ (kJ/mol)
Langmuir	0.999	slope	1.05	4.94 × 10^4^	−43.9
Freundlich	0.930	n	10.46	1.84 × 10^0^	−18.6
Temkin	0.944	a	−6.49	2.18 × 10^8^	−64.7
Frumkin	0.836	a	2.25	9.10 × 10^−7^	17.3

**Table 10:** Electrochemical parameters obtained for the mild steel in the presence and absence of the THTQ at different immersion times.

Medium	Time (h)	Rs (Ω.cm^2^)	R_p_ (Ω.cm^2^)	Q (µF.S ^n−1^)	n_dl_	C_dl_ (µF.cm^−2^)	ƞ_imp_ %
1M HCl	1/2	1.7	33.0	312.70	0.784	89.1	-
	1	1.6	26.4	364.90	0.810	122.7	-
	2	1.5	23.0	433.0	0.835	174.3	-
	4	1.5	21.4	627.00	0.834	267.0	-
	6	1.0	19.4	963.80	0.796	349.4	-
	12	1.2	10.4	949.2	0.764	419.5	-
THTQ	1/2	0.41	465.0	58.8	0.809	25.2	92.9
	1	0.29	540.0	35.48	0.829	15.58	93.9
	2	1.52	138.7	81.52	0.899	49.38	76.2
	4	1.35	130.4	103.9	0.883	58.9	74.7
	6	1.21	126.7	137.9	0.857	70.35	73.9
	12	1.11	115.0	207.3	0.837	101.0	71.3

**Table 11:** Weight percentage of elements obtained from EDX analysis.

Specimens	Fe	C	O	N	Cl
Mild steel in 1 M HCl	90.39	5.87	3.74	-	-
Mild steel in THTQ	86.13	9.50	2.29	1.38	0.70

**Table 12:** Global descriptors of reactivity for the protonated form of THTQ in aqueous phase.

Descriptors	E_HOMO_ (eV)	E_LUMO_ (eV)	ΔE_gap_ (eV)	Ƞ (eV)	σ (eV^−1^)	χ (eV)	ΔE_back-donation_	ΔN_Fe/110_
THTQ	−6.5444	−1.2754	5.2690	2.6345	0.3795	3.9099	−0.6586	0.1727

**Table 13:** E_ads_ for the studied molecular structure on Fe (110)/180 H_2_O system (all units in kcal/mol).

System	E_ads_	Inhibitor: dE_ad_/dN_i_	Rigid Adsorption Energy	Deformation Energy	H_2_O: dE_ad_/dN_i_	H_3_O^+^: dE_ad_/dN_i_	Cl^−^: dE_ad_/dN_i_
Fe_110_/THTQ	−7698.63	−276.32	−4068.72	−3629.91	−31.58	−172.83	−149.99

## Supplementary Materials

The following supporting information can be downloaded at: https://www.mdpi.com/article/10.3390/molecules28145340/s1, Figure S1: IR, 1H-NMR, 13C- NMR and MS spectrums of THTQ.
