# Supplementary figures and images for: Synthesis, Characterization, Antibacterial, Antifungal and Anticorrosion Activities of 1,2,4-Triazolo[1,5-a]quinazolinone
Source: Molecules. 2023 Jul 11;28(14):5340. doi: 10.3390/molecules28145340 (PMC10385296; doi:10.3390/molecules28145340)

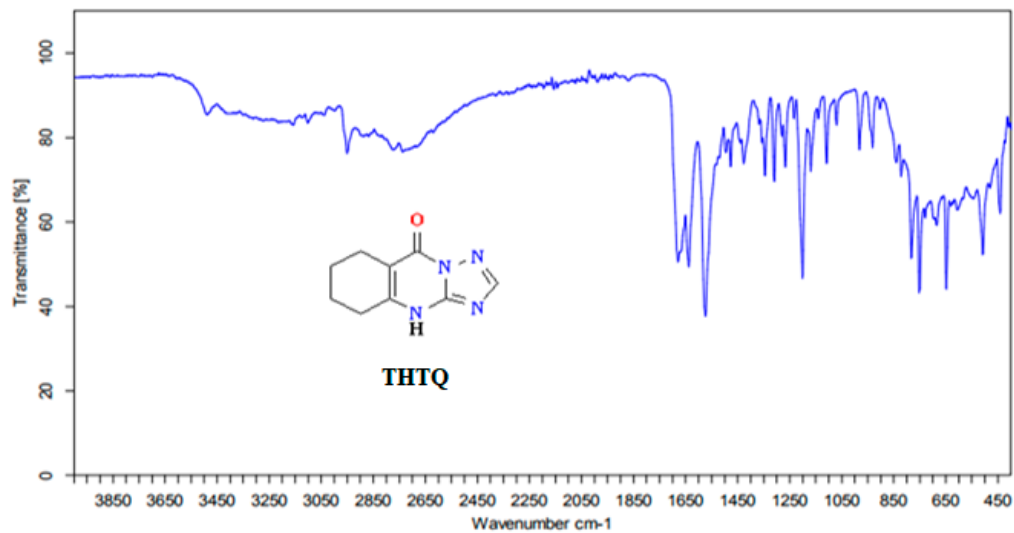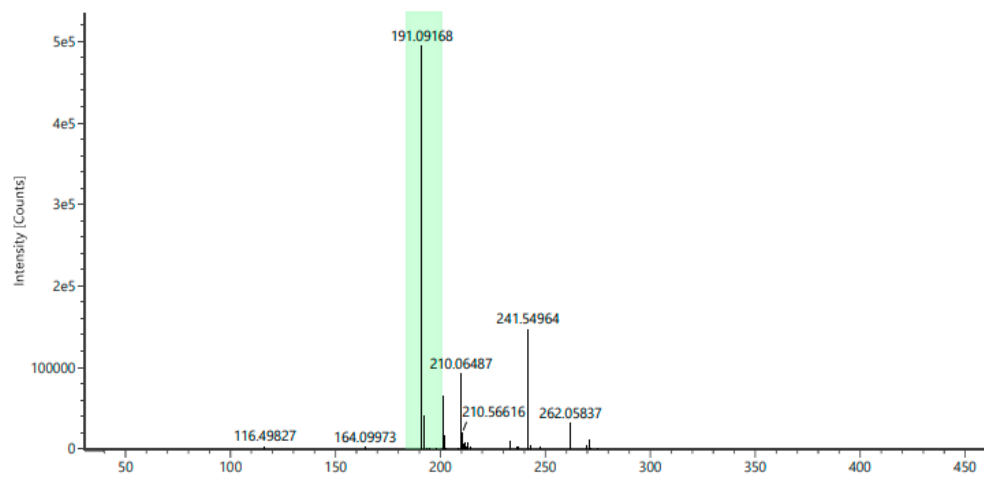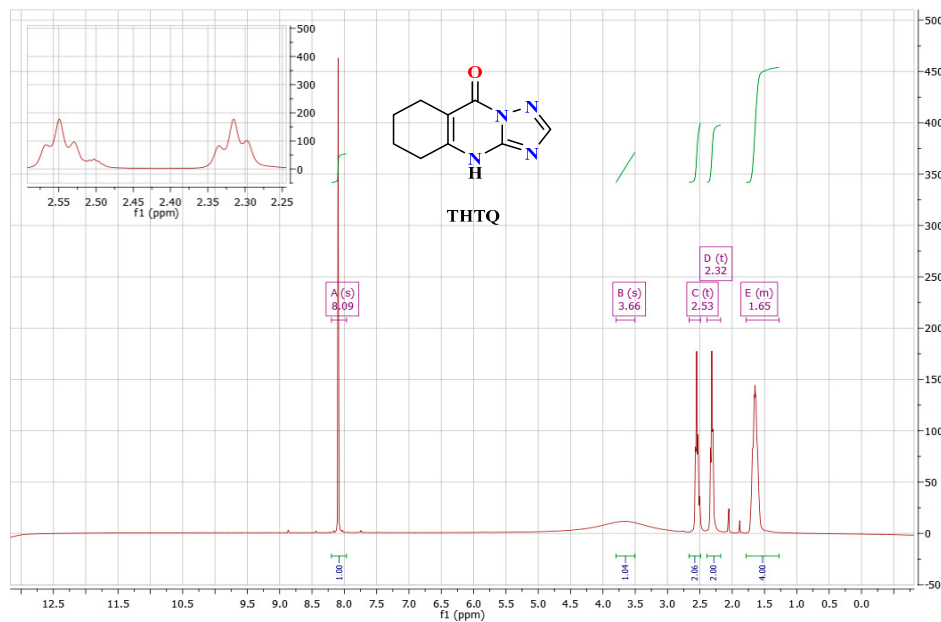

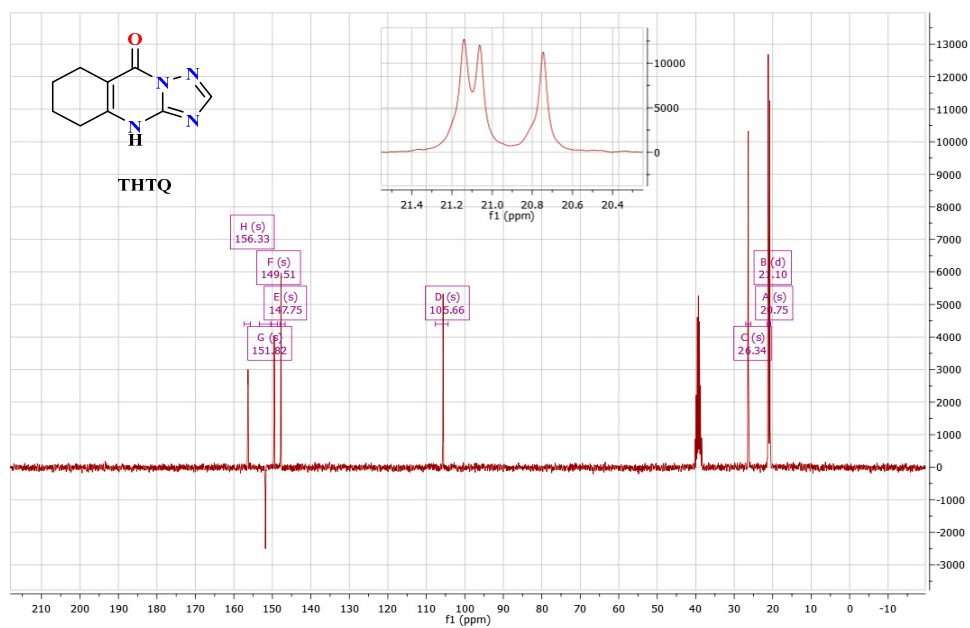

**Figure S1.** IR, <sup>1</sup>H-NMR, <sup>13</sup>C- NMR and MS spectra of THTQ

Supplement: Supplementary file 1 [file molecules-28-05340-s001.zip › molecules-2456870-supplementary.pdf]
